# Benzoxetes and Benzothietes - Heterocyclic Analogues of Benzocyclobutene 

**DOI:** 10.3390/molecules17021548

**Published:** 2012-02-07

**Authors:** Herbert Meier

**Affiliations:** Institute of Organic Chemistry, University of Mainz, Duesbergweg 10-14, D-55099 Mainz, Germany; Email: hmeier@mail.uni-mainz.de; Tel.: +49-6131-392-2605; Fax: +49-6131-392-5396

**Keywords:** cycloaddition, flash-vacuum-pyrolysis, photochemistry, ring closure, ring opening

## Abstract

Benzo-condensed four-ring heterocycles, such as benzoxetes **1** and benzothietes **3** represent multi-purpose starting compounds for the preparation of various higher heterocyclic ring systems. The thermal or photochemical valence isomerizations between the benzenoid forms **1**,**3** and the higher reactive *o*-quinoid structures **2**,**4** provide the basis for the synthetic applications. On the other hand, this valence isomerization impedes in particular the generation and storage of **1** because the thermal equilibrium **1** ⇆ **2** is completely on the side of **2**. Thus, the number of erroneous or questionable benzoxete structures published to date is surprisingly high. On the contrary, the thermal equilibrium **3** ⇆ **4** is on the side of the benzothietes **3**, which makes them easily accessible, especially by different flash vacuum pyrolysis techniques. The present article gives a survey of the preparations of **1** and **2**, and tries to stimulate their use in synthetic projects. Naphtho-condensed and higher condensed compounds and compounds with an exocyclic C=O or S=O double bond (lactones, thiolactones, sulfoxides and sulfones) are not covered in this article.

## 1. Introduction

**Figure 1:**
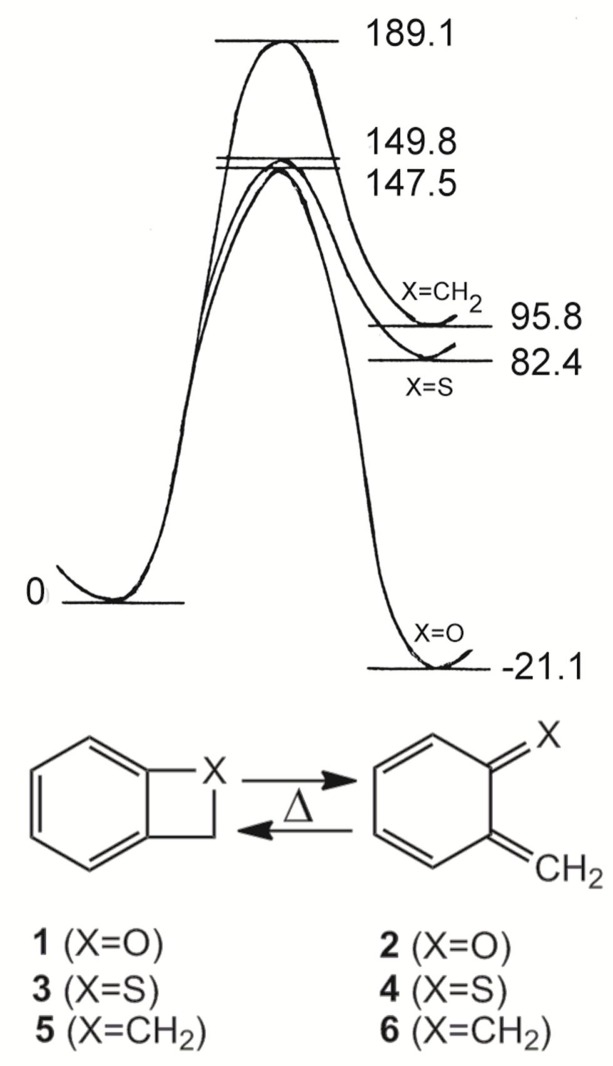
*Ab initio *calculation of the valence isomerizations **1** ⇆ **2** (X=O), **3** ⇆ **4** (X=S) and **5** ⇆ **6** (X=CH_2_). Energy differences in kJmol^−1^ [1].

2*H*-Benz[*b*]oxetes (**1**) and 2*H*-benzo[*b*]thietes (**3**), the heterocyclic analogues of benzocyclobutene (**5**) are highly interesting compounds because of their strained molecular structures which enables an easy thermal or photochemical ring opening to the *o*-quinoid valence isomers **2**, **4** and **6**, respectively. The latter 8π electron systems are reactive species which participate in a variety of addition and cycloaddition reactions. Figure 1 visualizes the thermal ring opening processes on the basis of *ab initio *calculations (HF/6-31G^**^) [1].

Early EHMO calculations revealed already an increasing tendency of the ring opening in the groundstate S_0_ as well as in the electronically excited singlet state S_1_ in the sequence CH_2 _< S < O [2]. Semiempirical quantum mechanics (MNDO) showed then that the ring opening **3** → **4** is an endothermic process [3]-as in the carbocyclic case **5** → **6**. The ring opening **1** → **2** however, is an exothermic reaction [4]:

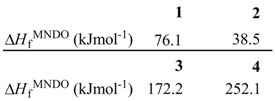


The corresponding enthalpy differences of −37.6 and +79.9 kJmol^−1^, respectively, agree very well to the results of earlier [1] or more recent *ab initio* calculations [5,6].

**Scheme 1:**
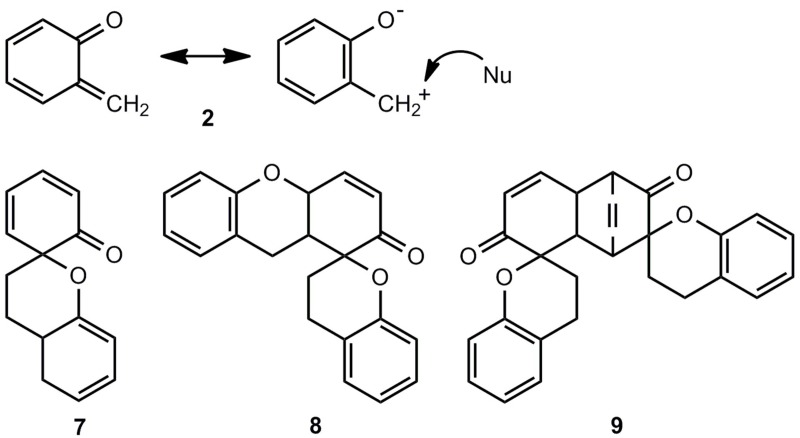
*o*-Quinone methide and its polycyclic oligomers.

The thermal ring opening of benzoxetes **1** to *o*-quinone methides **2** can occur already far below room temperature, whereas benzothietes **3** are stable at ambient temperatures and isomerize to *o*-thioquinone methides **4** in toluene at about 100 °C (ΔG^±^ = 120.0 kJmol^−1^) [3]. The calculated activation barriers, shown in Figure 1, are somewhat too high. These features demonstrate the essential difference between **1** and **3** in synthetic applications: The benzoxetes **1** cannot normally be stored, and they or better their open valence isomers have to be freshly prepared and reacted *in situ*. The benzothietes **3** on the other hand, can be stored and can be opened thermally or photochemically whenever needed [3]. Another difference concerns the chemical behavior of **1**/**2 **and **3**/**4** in the absence of reaction partners such as nucleophiles or dienophiles. *o*-Quinone methide **2** forms dimers, trimers and tetramers by repetitive Diels-Alder reactions [7,8,9,10,11,12]. Scheme 1 shows the [2π+4π]- or better [2π+8] cycloadditions to the dimer **7**. Apart from the exocyclic CC double bond, one of the endocyclic double bonds of **2** can also represent the 2π component [12]. The hetero-Diels-Alder adduct **7** can enter then a further [2π+8] cycloaddition to yield the trimer **8**, but **7** can also dimerize in a normal Diels-Alder reaction to the tetramer **9** [11].

**Scheme 2:**
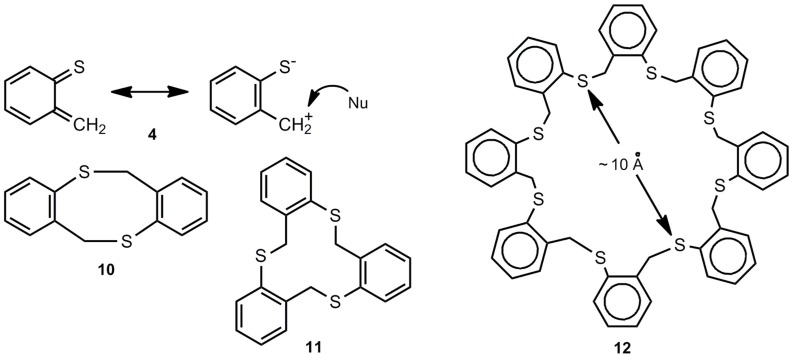
*o*-Thioquinone methide and its cyclooligomers.

*o*-Thioquinone methide **4** behaves completely different (Scheme 2). It generates the [8π+8π] cyclodimer **10** and small amounts of higher cyclooligomers (n = 3-8) among which the cyclotrimer **11** and the cyclooctamer **11** are major components [3,9,13,14,15]; compounds **11** and **12** represent interesting thiocrown ethers.

## 2. Benzoxetes

### 2.1. Isolation of Benzoxetes

**Scheme 3:**
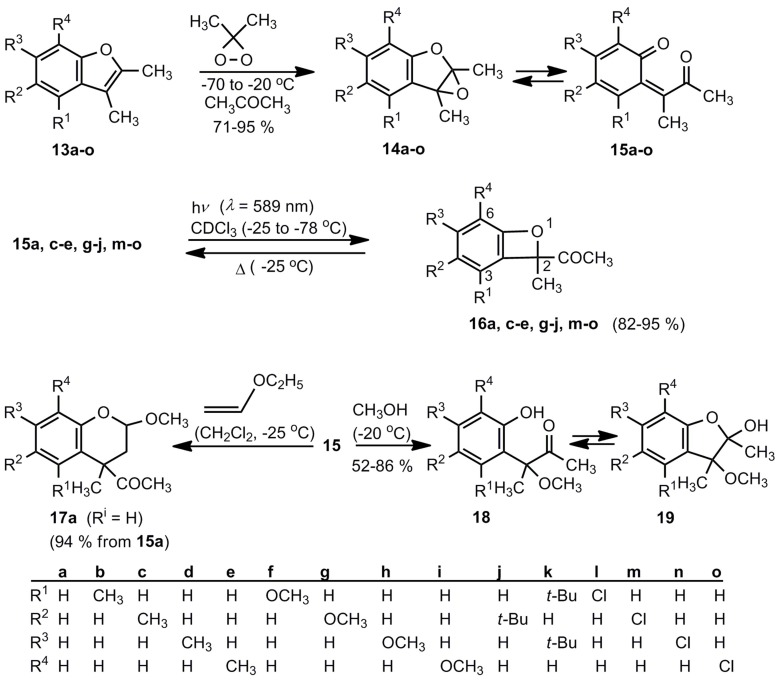
The first successful approach to benzoxetes.

The first isolable benzoxetes were obtained by Adam *et al.* [12,16]. Scheme 3 demonstrates the mode of preparation. Low temperature oxidations of benzofurans **13a**-**o** by dimethyldioxirane afford mixtures of the epoxides **14a**-**o** and the *o*-quinone methides **15a**-**o** in high yields. The ratio of the **14/15** equilibrium depends on the substituents varying from almost pure **14f** to almost pure **15h**. All these trienediones **15** have (*Z*)-configurations. Irradiation (*λ *= 589 nm) of the **14/15** mixtures between −25 and −78 °C yields then in most cases the desired benzoxetes (conversion ≥ 95%). Exceptions are the systems **14/15b**,**f**,**k**,**l** (R^1^ ≠ H). Low-temperature irradiations are necessary because the *o*-quinone methides **15** can isomerize above 0 °C by 1.5-H shifts to phenols [12]. Moreover, the benzoxetes **16** revert thermally to the valence isomers **14/15**. The Cl-containing compounds **16m**-**o** exist at −10 °C for approximately 1h, the OCH_3_ systems **16g**-**i** are even more labile. The resulting *o*-quinone methides can then oligomerize, as discussed above. In the presence of enol ethers, compounds **15** yield 3,4-dihydro-2*H*-benzopyrans, as the example **17a** reveals, and in the presence of methanol a tautomeric mixture of the hydroxyketones **18** and their hemiacetals **19** is obtained [12].

The benzoxetes 16 were characterized by their ^1^H- and ^13^C-NMR spectra at low temperatures. The quaternary carbon atoms of the oxete ring show typical ^13^C chemical shifts: Δ (C-2) = 102 ± 2, δ (C-2a) = 132 ± 3 and δ (C-6a) = 162 ± 3 ppm [12,16].

**Figure 2:**
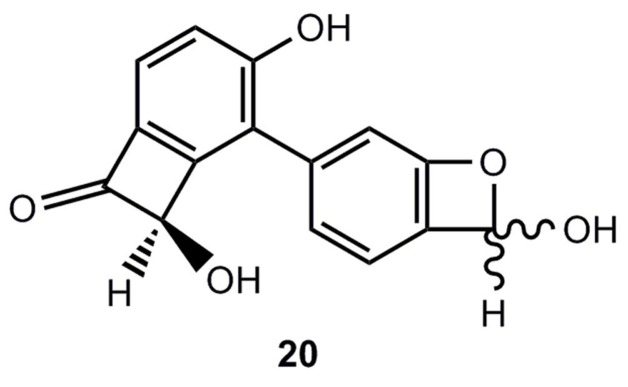
A natural product, for which a benzoxete structure was postulated.

Recently a Chinese research group [17] reported the isolation of a 5-aryl-2-hydroxybenzoxete **20** from the stem of *Caesalpinia decapetala* (Figure 2). Although the structure was carefully studied by NMR including HMBC measurements, the stability of **20** raises some doubts about the validity of the proposed structure [18,19,20].

### 2.2. Matrix Isolation of Benzoxetes

**Scheme 4:**
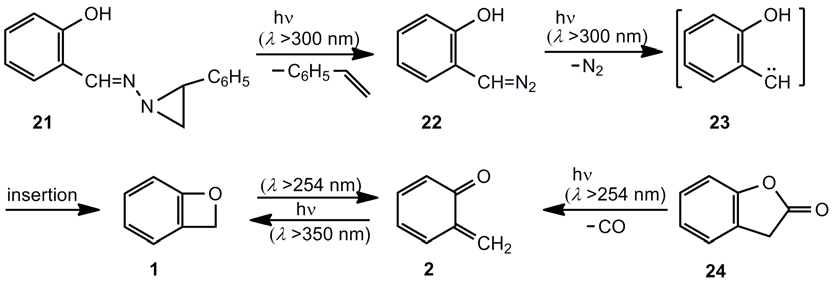
Generation of unsubstituted benzoxete.

Unsubstituted benzoxete 1, the parent compound, was first obtained by Tomioka *et al.* (Scheme 4) [1,21]. The masked diazo compound 21, developed by Eschenmoser [22], was used to produce the carbene 23 via the diazo system 22 at 10 K in an Ar matrix. The IR spectra revealed the formation of *o*-quinone methide (2) together with its valence isomer, benzoxete 1. Benzofuranone 24 provides another successful entry to the wavelength-dependent ratio 1/2.

**Scheme 5:**
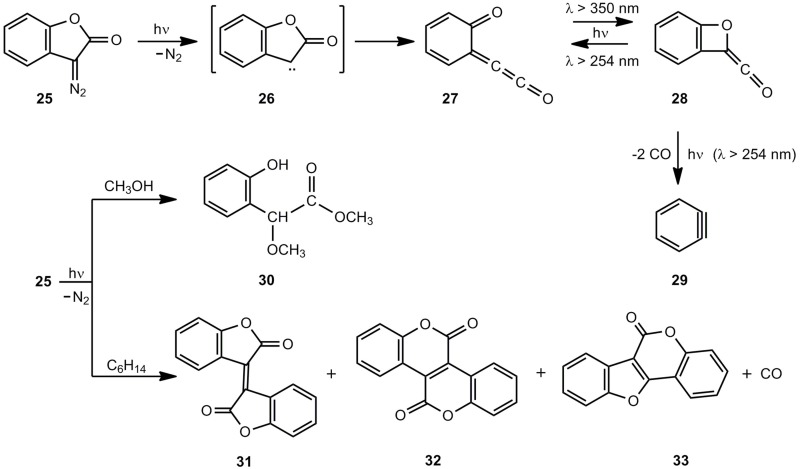
Photolysis of 3-diazo-2(3*H*)-benzofuranone.

Chapman *et al.* [23] studied the cyclic diazo compound 25 (Scheme 5). According to IR and UV measurements, irradiation at 8 K in an Ar matrix generated carbene 26, which formed 27 and its photoproduct 28 [23,24]. The 27/28 ratio depends on the wavelength applied; however, an extended irradiation at 254 nm yields the 1,2-didehydrobenzene 29. Irradiation of 25 in methanol at ambient temperatures furnishes the ester 30 [25] and irradiation in acidic aqueous solutions gives the corresponding carboxylic acid and 3-hydroxy-3*H*-benzofuran-2-one [24]. In hexane, the intermediate primary photoproduct 27 dimerizes to isoxindigo 31, small amounts of bislactone 32 and coumestan 33, the decarbonylated product [24]. 

**Scheme 6:**
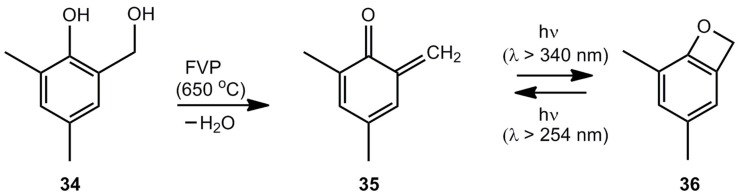
Generation of 4,6-dimethylbenzoxete.

Wentrup *et al.* [8] generated the equilibrium mixture of 1 and 2 by flash-vacuum-pyrolysis (FVP) of benzofuran-2-one (24) or 2-(hydroxymethyl)phenol followed by the photochemical cyclization 2 → 1. Warm-up experiments demonstrated that benzoxete 1 is stable up to at least 155 K. Surprisingly, methyl substituents on the benzene ring stabilize the benzoxete system 36 significantly (Scheme 6).

**Scheme 7:**
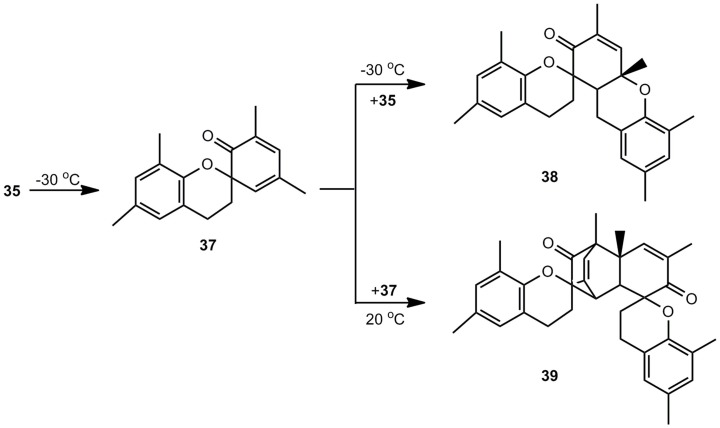
Polycyclic oligomers of 4,6-dimethylbenzoxete.

4,6-Dimethylbenzoxete (**36**), obtained at 7.6 K in an Ar matrix, was characterized by IR spectroscopy. In the presence of water, the dihydroxy compound 34 is recovered, in the absence of nucleophiles dimer **37**, trimer **38** and tetramer **39** (Scheme 7) are obtained [8].

Trimer **38**, subjected to FVP at 850 °C, is a good precursor for *o*-quinone methide **35**. The dehydration of 2-hydroxymethylphenols by FVP, shown in Scheme 6, can also be achieved by irradiation [5].

### 2.3. Benzoxetes as Intermediates

**Scheme 8:**
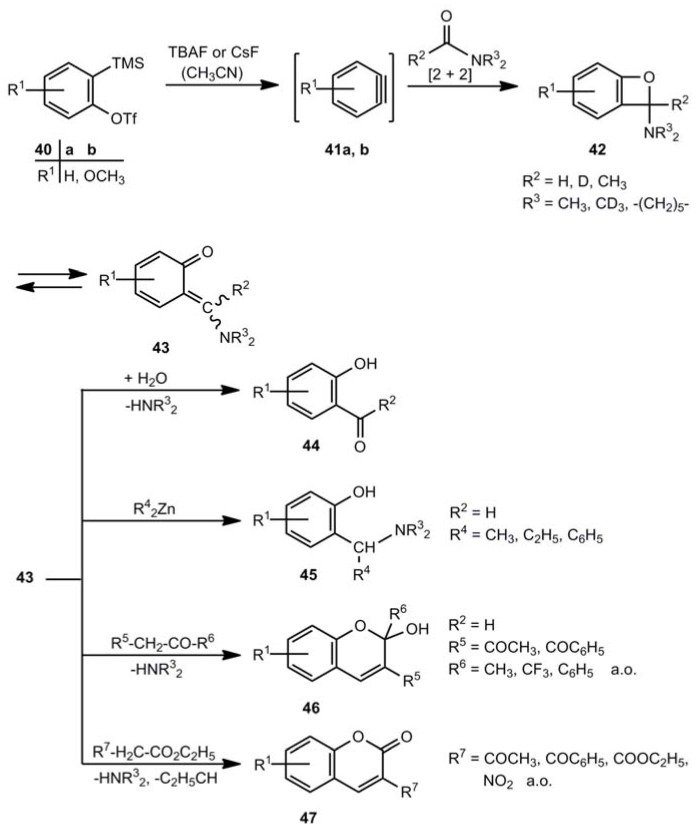
Benzoxetes as intermediate in the reaction of arynes and carboxamides.

Benzoxetes can be intermediates in various reactions. A quite new example is shown in Scheme 8 [26,27]. Benzyne or other arynes react with *N*,*N*-dialkylformamides or *N*,*N*-dialkylacetamides. Treatment of **40** with tributylammonium or cesium fluoride in acetonitrile generates the arynes **41**, which undergo with carboxylic acid amides [2+2] cycloadditions to the benzoxetes **42**. Their corresponding *o*-quinone methides **43** can be trapped by water to afford **44** [26], or with zinc organic compounds to form **45** [27], or by reactive methylene components to generate **46** [26] and **47** [26], respectively. The yields of **44-47** are moderate to good.

**Scheme 9:**
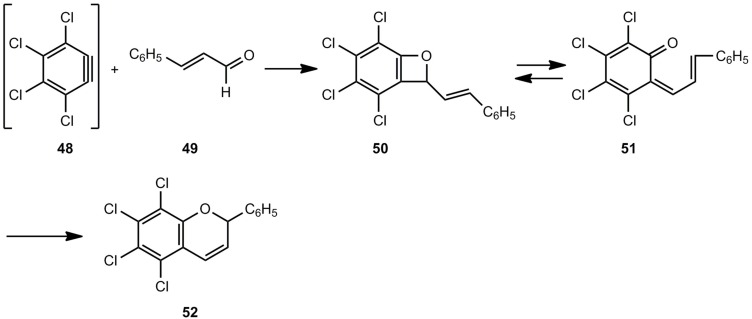
3,4,5,6-Tetrachloro-2-styrylbenzoxete as intermediate in the reaction of an aryne and cinnamaldehyde.

A related [2+2] cycloaddition was already found in the early seventies [28,29]. The dehydrobenzene 48 reacted with α,β-unsaturated aldehydes, such as cinnamaldehyde (49), to yield 5,6,7,8-tetrachloroflav-3-ene (52) via the benzoxete 50 and its valence isomer 51 (Scheme 9).

**Scheme 10:**
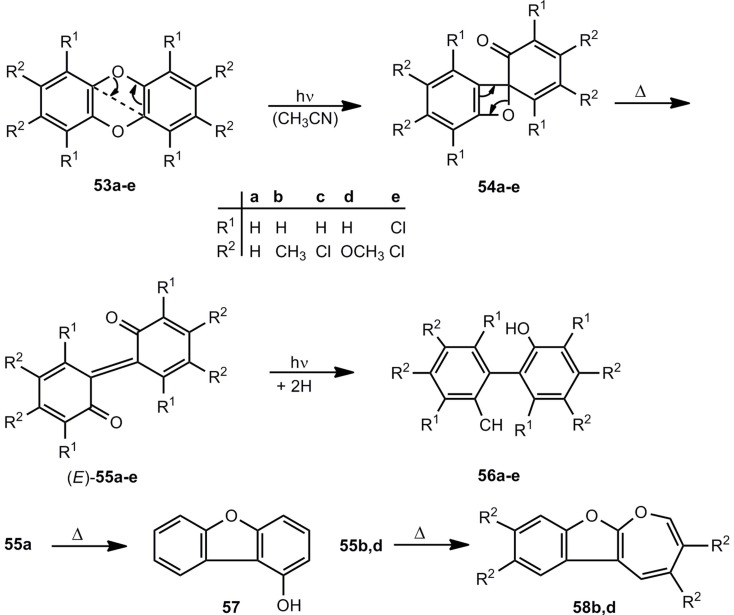
Photolysis of dibenzo [1,4] dioxins.

According to UV reaction spectra of the photolysis of dibenzo [1,4] dioxins 53, the spiro compounds 54 were postulated as intermediates on the route to the 2,2'-biphenylquinones 55 (Scheme 10). The initial aryl ether cleavage 53 → 54 is followed at room temperature by the thermal valence isomerization to 55. Under steady-state irradiation conditions, the quinones 55 undergo in the excited state a hydrogen abstraction from the solvent. Thus, the 2,2'-dihydroxybiphenyls 56 are generated in reasonable yields [30].

In the dark, **55a** and 2,2'-biphenylquinones with electron-withdrawing substitutents rearrange to 1-hydroxydibenzofurans **57**, whereas the 2,2'-biphenylquinones **55b**,**d** with electron-releasing substituents form oxepino[2,3-*b*]benzofurans (**58**) [30].

### 2.4. Erroneous Benzoxete Structures

Apart from the photochemically generated spiro compounds **54a**-**e**, many spiro[2,4-cyclohexadiene-1,8'-[7]oxabicyclo[4.2.0]octa-(1,3,5)-trien]-6-ones **54'f**-**o** [31,32,33,34,35,36,37,38,39,40,41,42,43,44,45] and **54″p**,**q** [34] (Table 1) have been postulated as thermal reaction products. Table 1 provides a survey over these sterically hindered systems, whose benzoxete structures were proved to be wrong.

**Table 1:** Erroneous benzoxete structures **54'** and **54**″. 
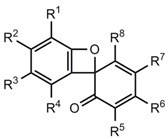

Compound	R^1^	R^2^	R^3^	R^4^	R^5^	R^6^	R^7^	R^8^	References
**54'f**	*t*-Bu	H	*t*-Bu	H	*t*-Bu	H	*t*-Bu	H	[31,36,37,39,42,43,44,47]
**54'g**	*t*-Bu	H	OMe	H	*t*-Bu	H	OMe	H	[32,33,34,46,47]
**54'h**	*t*-Bu	H	CMe_2_Et	H	*t*-Bu	H	CMe_2_Et	H	[35]
**54'i**	CMe_2_Et	H	*t*-Bu	H	CMe_2_Et	H	*t*-Bu	H	[35]
**54'j**	CMe_2_Et	H	CMe_2_Et	H	CMe_2_Et	H	CMe_2_Et	H	[36,40,42,47]
**54'k**	*t*-Bu	H	CPh_3_	H	*t*-Bu	H	CPh_3_	H	[41,42,47]
**54'l**	*t*-Bu	H	OC_6_H_4_-COOEt	H	*t*-Bu	H	OC_6_H_4_-	H	[38]
**54'm**	*t*-Bu	H	*t*-Bu	Me	*t*-Bu	H	*COOEt*	Me	[45,47]
**54'n**	*t*-Bu	H	*t*-Bu	Cl	*t*-Bu	H	*t* -Bu	Cl	[45,47]
**54'o**	*t*-Bu	Cl	OMe	Cl	*t*-Bu	Cl	*t* -Bu	Cl	[45,47]
**54″p**	*t*-Bu	H	OMe	H	*t*-Bu	H	OMe	OMe	[34,49]
**54″q**	*t*-Bu	H		H	*t*-Bu	H	OMe	OEt	[34,49]

**Scheme 11:**
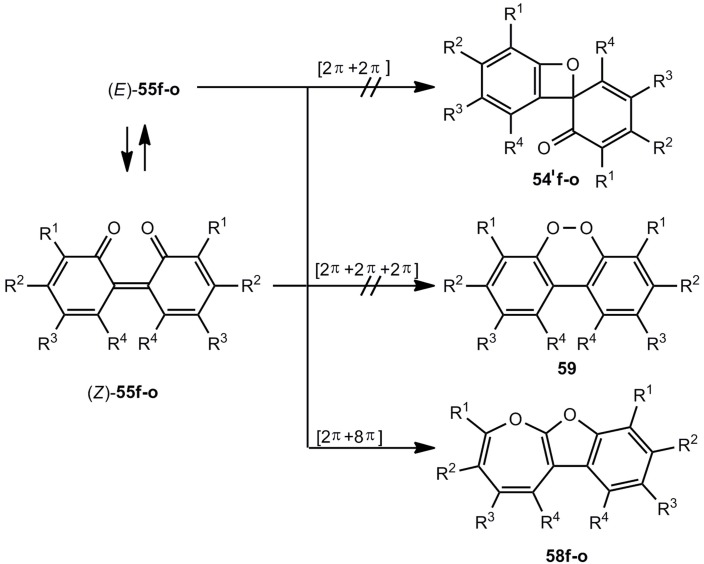
Valence isomers of 2,2'-biphenylquinones.

The oxidation of phenols or biphenols leads to 2,2'-biphenylquinones **55** (Scheme 11) for which three types of electrocyclic ring closure reactions can be conceived. The formation of dibenzo[c,e,^1^,^2^]dioxins **59** [46] was discounted and the generation of sterically hindered benzoxetes **54'f**-**o** claimed [31,32,33,34,35,36,37,38,39,40,41,42,43,44,45,46]. However, it turned out on the basis of NMR studies and a crystal structure analysis of **58o** [47,48], that the real structures of **54'f**-**o** are oxepino[2,3-*b*]benzofurans **58f**-**o** [47,48].

**Figure 3:**
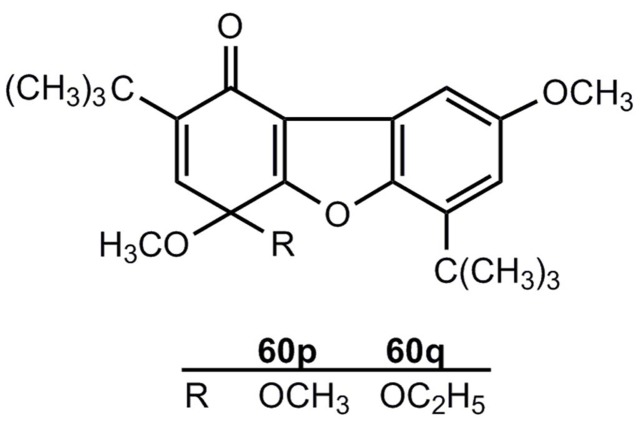
1(4*H*)-Dibenzofuranones.

In contrast to the photochemical generation shown in Scheme 10, there is no thermal route **55** → **54**'. The compounds **54″p**,**q** are reaction products of **54'g** and have the structures **60p**,**q** [48] (Figure 3).

**Scheme 12:**
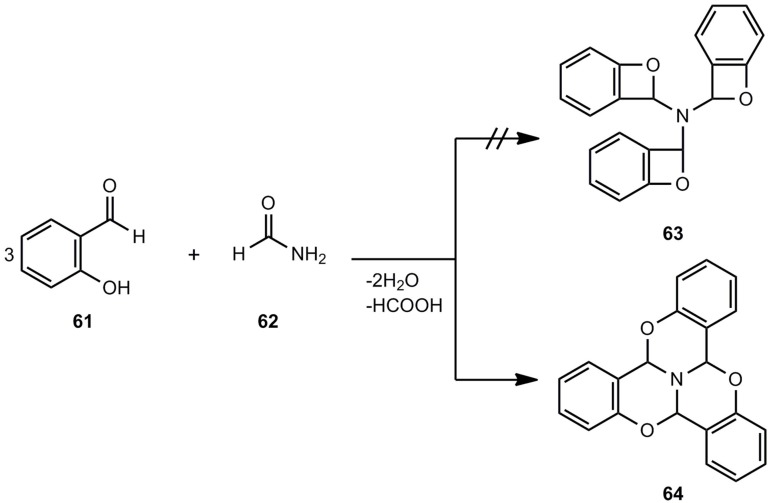
Reaction of salicylic aldehyde and formamide.

Out of a series of older references [50,51,52,53,54,55,56,57] on alleged benzoxetes, just one shall be discussed here, namely the work of Mastagli *et al.* [54], which provides an interesting polycyclic system **64** instead of a threefold benzoxete (**63**) (Scheme 12). The reaction of salicylic aldehyde (**61)** and formamide (**62**) yields the ring system **64**, a tribenzotrioxaazaphenalene [58].

## 3. Benzothietes

### 3.1. Preparation of Benzothietes by Ring Contraction Reactions

**Scheme 13:**
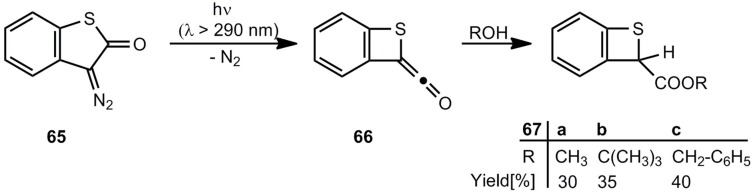
Photochemical Wolff rearrangement of 3-diazobenzo[*b*]thiophen-2(3*H*)-one.

The first successful synthesis of benzothietes was published in 1976 by Meier *et al.* The Wolff rearrangement of 3-diazobenzo[*b*]thiophen-2(3*H*)-one (65) yields the ketene 66 and in the presence of alcohols the corresponding esters 67a-c (Scheme 13) [59,60].

**Scheme 14:**
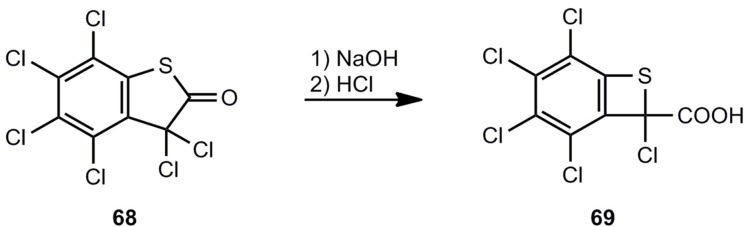
Ring contraction of perchlorobenzo[*b*]thiophen-2(3*H*)-one.

A kind of Favorsky rearrangement can be used for the ring contraction of the hexachloro compound **68** to afford the benzothiete derivative **69** (Scheme 14) [61].

### 3.2. Benzothietes by Cycloelimination Reactions

**Scheme 15:**
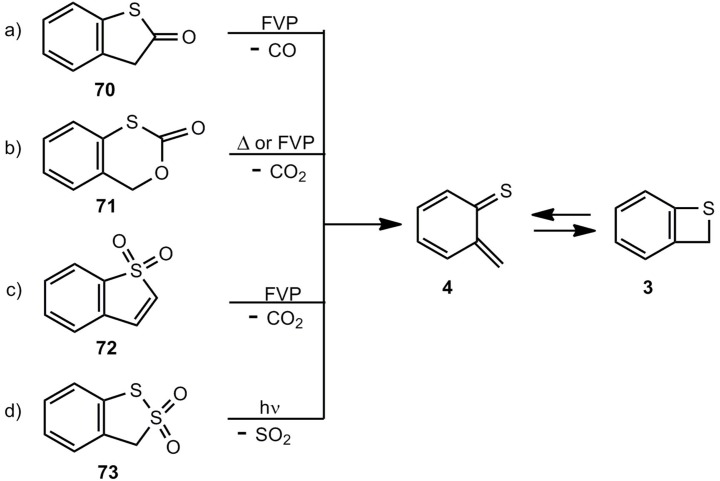
Thermal or photochemical cycloelimination reactions leading to benzothiete.

Several cycloelimination reactions of CO, CO_2_ or SO_2_ can be applied for the generation of benzothiete (Scheme 15). The parent compound **3** could be originally obtained by a multi-step degradation of **67b** [3,59], but each of the elimination routes a)-c), shown in Scheme 15, provides a much easier route.

**Scheme 16:**
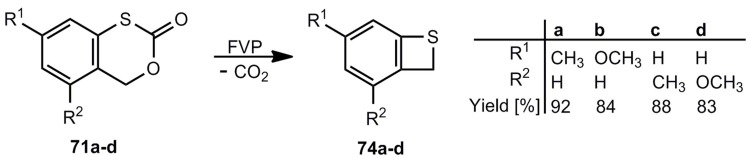
Preparation of substituted benzothietes by flash-vacuum-pyrolysis.

The decarbonylation of **70** by flash-vacuum-pyrolysis (FVP) [14,62] and the decarboxylation of **71** by thermolysis in solution or FVP [63,64] look straightforward. Interestingly, the sulfone **72** does not eliminate SO_2_, and after a rearrangement CO_2_ is split off [65,66]. In cold traps, **3** can be isolated in all these cases in yields up to 90%. The photodesulfonylation of **73** in benzene however, can only be used for trapping reactions of *o*-thiobenzoquinone methide **4** [67]. Substituted benzothietes (Scheme 16) can be obtained in high yields by FVP of the corresponding benzoxathiinones **71** [64].

### 3.3. Benzothietes by Cyclization Reactions

**Scheme 17:**
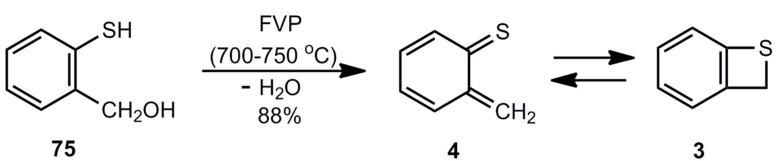
Preparation of unsubstituted benzothiete by flash-vacuum-pyrolysis of 2-mercaptobenzyl alcohol.

Boekelheide *et al.* [9] developed the preparation of benzothiete (3) by FVP of 2-mercaptobenzyl alcohol (75) (Scheme 17).

Fifty g of **3** per hour can be obtained in a suitable flow device [3]. Side products are not formed and the amount of unreacted starting material can be reduced by increasing the contact time [68]. Instead of the hydroxyl compound, *o*-chloromethylthiophenol can be used, too [3].

**Scheme 18:**
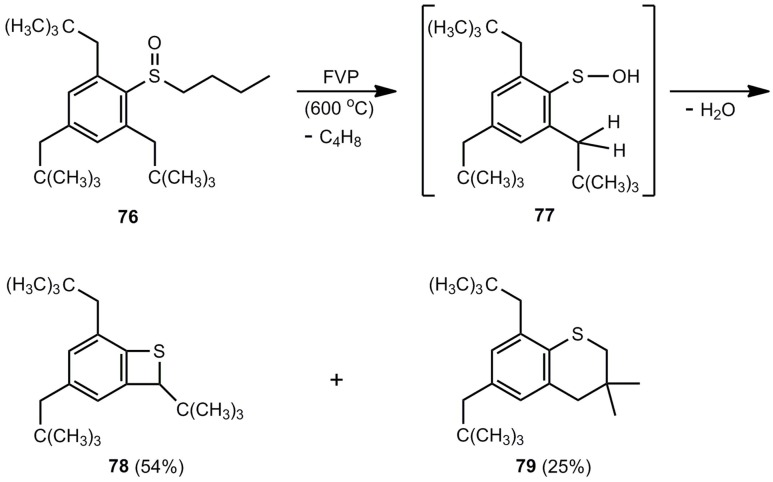
Flash-vacuum-pyrolysis of 2-butylsulfonyl-1,3,5-tris(2,2-dimethylpropyl)benzene.

Another 1,4-elimination was studied with benzene sulfenic acid **77**, an intermediate in the FVP of **76** (Scheme 18). The elimination of H_2_O can occur with the methylene group or with one of the methyl groups. Therefore, benzothiete **78** and benzo [*b*]thiopyran **79** are obtained [69].

**Scheme 19:**
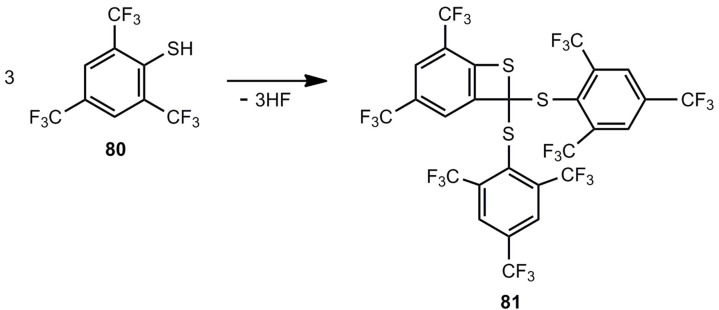
Elimination of hydrogen fluoride for the preparation of a highly substituted benzothiete.

An unusual cyclization reaction was observed for 2,4,6-tris(trifluoromethyl)thiophenol [70]. The threefold elimination of HF led to benzothiete **81**, whose structure was confirmed by a crystal structure analysis (Scheme 19). The reaction was performed in the presence of Ga(CH_3_)_3_, whose role is not established.

**Figure 4:**
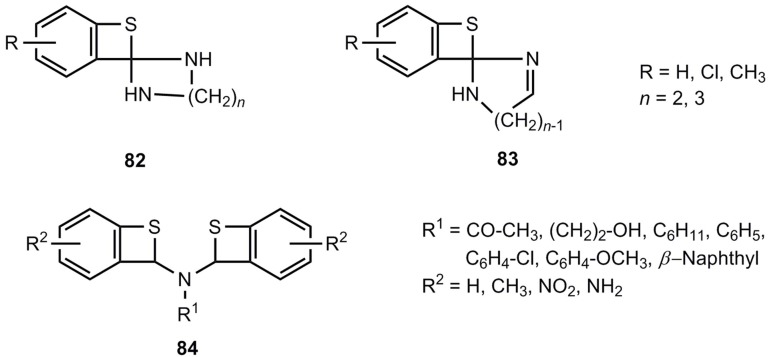
Postulated benzothietes with amino substituents.

A series of spiro-compounds **82** and **83** were reported as reaction products of 2-chloro-benzaldehydes, *α*,*w*-diamines and sulfur [71,72]. However, since the corresponding benzoxetes [50] certainly have different structures, a reinvestigation of **82** and **83** seems to be advisable. The same is true for the compounds 84 [73,74,75] (Figure 4).

### 3.4. Benzothietes by Cycloaddition Reactions

**Scheme 20:**
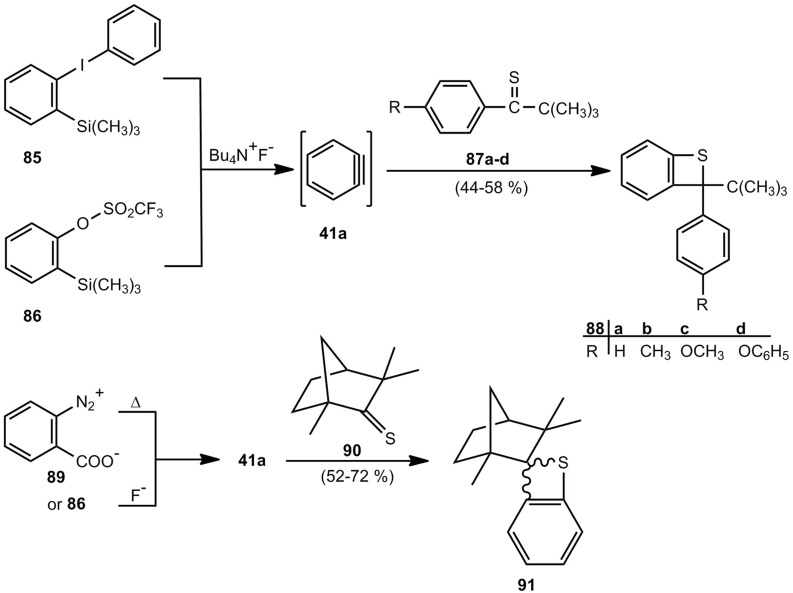
Reaction of 1,2-didehydrobenzene and thioketones.

A relatively new synthesis of benzothietes is based on [2+2] cycloaddition reactions of 1,2-didehydrobenzene (**41a**) and thiocarbonyl compounds. Whereas the reaction of **41a**, obtained from *o*‑benzenediazonium carboxylate and thiophosgene led to 2,2-dichlorobenzothiete among a vast mixture of products [76], the reaction of **41a** and sterically hindered and/or electronically stabilized thioketones **87a-d** [77,78] or **90** [77] is reasonably efficient (Scheme 20).

The benzothietes **88a**-**d** are racemates, but thiofenchone (**90**) however, gives the diastereomeric cycloadducts **91**. The components show a ratio of 7:1 in favor of the system with S in *exo*-position [77].

### 3.5. Synthetic Applications of Benzothietes

**Scheme 21:**
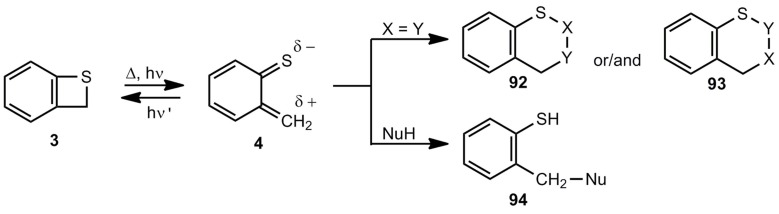
Addition and cycloaddition reactions of benzothiete/*o*-thioquinone methide.

In contrast to the less stable benzoxetes, benzothietes are very useful for the preparation of S-heterocycles and benzene derivatives with SR groups. Two important reaction types have to be mentioned here, namely the cycloaddition of the corresponding thioquinone methides **4** as 8π components with 2π (or 4π) components and the addition of nucleophiles to **4** (Scheme 21). 

Table 2 gives a survey over the [8π/2π] and [8π/8π] cycloadditions of benzothiete (3) leading to the heterocyclic scaffolds **95-110**.

**Table 2:** [8π/2π]- and [8π/8π] Cycloaddition reactions of benzothietes.

[2π/8π]Components	Reaction Products		References
C=C	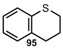	3,4-Dihydro-2*H*-benzothiopyran(Thiochroman)	[14, 63, 79, 80, 81, 82, 83, 84]
Alkenes			
Allenes			[85]
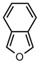 Benzo[*c*]furans	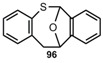	11,12-Dihydro-6,11-epoxy-6*H*-dibenzo[*b*,*f*] thiocine	[84]
CºC	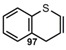	4*H*-1-Benzothiopyran(4*H*-Thiochromen)	[14]
Alkynes			
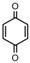 1,4-Quinones (Oxidation)	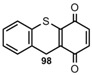	9*H*-Thioxanthene-1,4-dione	[82]
C=N	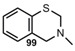	3,4-Dihydro-2*H*-1,3-benzothiazine	
Azomethines			[81,86,87]
Azines			[81]
Ketenimines			[85]
Carbodiimides			[85]
Oximes and their O-derivatives			[81,88]
C=O	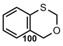	3,1-Benzoxathian	[87,89]
Carbonyl Compounds			
C=S	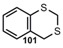	1,3-Benzodithian	[90]
Thiocarbonyl Compounds			
CºN	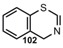	4*H*-Benzo[*e*,^1^, ^3^]thiazine	[91]
Nitriles			
N=C=S	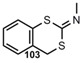 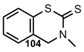	2-Imino-4*H*-1,3-Benzodithiin3,4-Dihydro-2*H*-1,3-Benzothiazine-2-thione	[91]
Isothiocyanates			
N=N	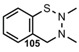	3,4-Dihydro-2*H*-1,2,3-benzothiadiazine	[87]
Azo Compounds			
N=O	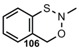 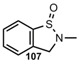	3,1,2-Benzoxathiazine2,3-Dihydro-1,2-benzo=thiazol-1-oxide	[87, 92]
Nitroso Compounds			
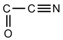 2-Oxonitriles	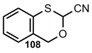	3,1-Benzoxathian-2-carbonitrile	[91]
N=S=O	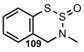	1,2,3-Benzodithiazine-2-oxide	[85]
N-Sulfinylamines			
P=S	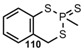	4*H*-1,3,2-Benzodithia=phosphorin-2-sulfide	[3]
Lawesson`s reagent			

**Figure 5:**
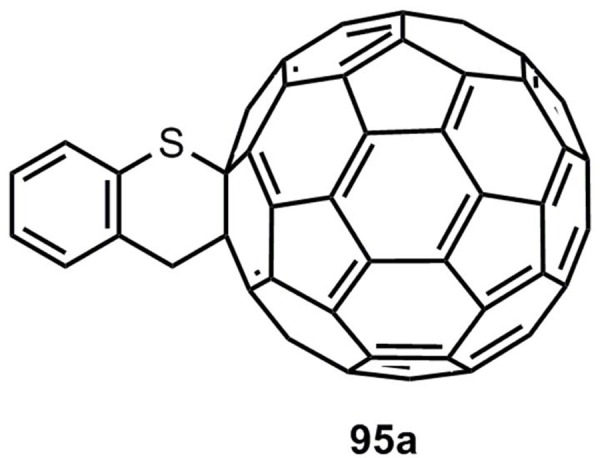
Adduct of benzothiete and fullerene C_60_.

The reactivity and the regio- and stereoselectivity of all these reactions have been discussed in detail [3,93]. Finally the cycloaddition of benzothiete and fullerene C_60_ shall be mentioned. The monoadduct **95a** is generated in a yield of 54% [94] (Figure 5).

**Figure 6:**
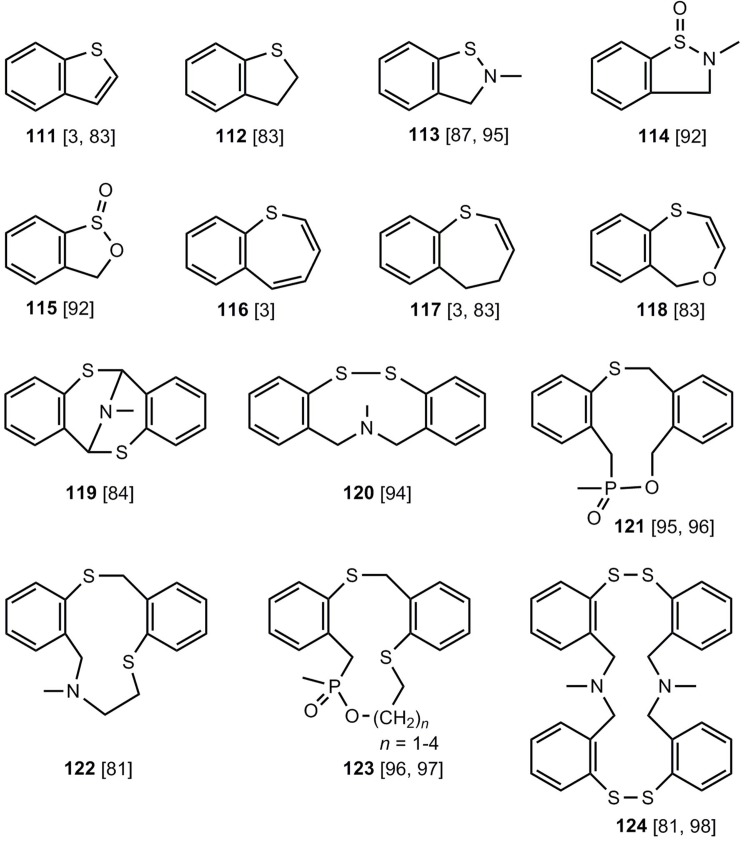
Further sulfur-heterocycles, which can be obtained from benzothiete.

In addition to the formation of six- and eight-membered rings, five-, seven-, nine- and eleven-membered heterocycles **111-122** and macrocyclic systems **123**, **124** (Figure 6) can be synthesized by applying benzothietes.

Dimerization of benzothiete **3 ⇆ 4** → **10** competes in all these reactions of 3 with dienophiles and nucleophiles. The less reactive the reaction partner is, the higher is the amount of 10. However, the portion of **10** is not completely lost, because FVP of 10 can lead back to **3**.

## 4. Conclusions

Benzoxetes **1** and benzothietes **3** seem to be very similar compounds. Both have low activation barriers for the opening of the four-membered rings, but the thermal equilibrium is for the benzoxetes (1) on the side of the *o*-quinone methides **2**, whereas it is for the *o*-thioquinone methides **4** on the side of the benzothietes **3**.

The different energetic situation has far-reaching consequences for the preparation of these compounds and their applications. Very few examples of substituted benzoxetes **1** have been obtained by photochemical reactions at low temperatures. The number of questionable or erroneous benzoxete structures is surprisingly high. It is much easier to generate and apply their open valence isomers, the *o*-quinone methides **2** [99]. The benzothietes **3**, on the other hand, can be prepared by various reactions including ring contractions, cycloeliminations, cyclizations and cycloadditions. The simple access to benzothietes **3** and their high reactivity in addition and cycloaddition reactions offers a variety of applications in the synthesis of benzo-condensed *S*-heterocycles (5- to 11-membered rings and macrocycles). Table 2 summarizes for example the access to 14 different heterocyclic 6-ring systems. Another promising application can be based on the optical switching **3** ⇆ **4**. Photokinetical studies [100] encourage such an application.

## References

[1] Tomioka H., Matsushita T. (1997). Benzoxetene. Direct observation and theoretical studies. Chem. Lett..

[2] Kolshorn H., Meier H. (1977). EHT calculations of valence isomerization between benzenoids and *o*-quinoidal systems. Z. Naturforsch..

[3] Meier H., Mayer A., Gröschl D. (1994). Benzothietes-Versatile synthons for the preparation of heterocycles. Sulfur Rep..

[4] Eckes H.-L. (1990). Nucleophilic addition and cycloaddition reactions of benzothiete. Doctoral Dissertation.

[5] Arumugam S., Popik V.V. (2009). Photochemical generation and the reactivity of *o*-naphthoquinone methides in aqueous solutions. J. Am. Chem. Soc..

[6] Shanks D., Frisell H., Ottoson H., Engman L. (2006). Design principles for α-tocopherol analogues. Org. Biomol. Chem..

[7] Latulle M., Guenot P., Ripoll J.-L. (1991). The syntheses of 6-methylene-2,4-cyclohexadien-1-imine and related *o*-quinonoids by FVT of 1-heter*o*-1,2,3,4-tetrahydronaphthalenes. Tetrahedron Lett..

[8] Quiao G.G.H., Lenghaus K., Solomon D.H., Reisinger A., Bytheway I., Wentrup C. (1998). 4,6-Dimethyl-*o*-quinone methide and 4,6-dimethylbenzoxete. J. Org. Chem..

[9] Mao Y.-L., Boekelheide V. (1980). Benzocyclobutene-*o*-Xylylene valence tautomerization: Oxygen and sulfur analogs. Proc. Natl. Acad. Sci. USA.

[10] Cavitt S.B., Sarrafizadeh H., Gardner P.D. (1962). The structure of *o*-quinone methide trimer. J. Org. Chem..

[11] Faure R., Thomas-David G., Bartnik R., Cebulska Z., Graca E., Lasniak S. (1991). Tetramer of *o*-methylenequinone. Bull. Soc. Chim. France.

[12] Adam W., Hadjiarapoglou L., Peters K., Sauter M. (1993). Dimethyldioxirane epoxidation of benzofurans: reversible thermal and photochemical valence isomerization between benzofuran epoxides, quinone methides, and benzoxetenes. J. Am. Chem. Soc..

[13] Van Tilborg W.J.M., Plomp R. (1977). Flash vapor-phase pyrolysis of thiophene 1,1-dioxides. Recl. Trav. Chim. Pays-Bas.

[14] Kanakarajan K., Meier H. (1983). Cycloaddition reactions of benzothiete. J. Org. Chem..

[15] Van Tilborg W.J.M., Plomp R. (1977). Synthesis of benzothiete from benzo[*b*]thiophene 1,1-dioxide. J. Chem. Soc. Chem. Commun..

[16] Adam W., Sauter M., Zuenkler C. (1994). Preparation of 2*H*-benzoxetes by photoinduced [2 + 2] cyclo addition of quinone methides, accessible by dimethyldioxirane (DMD) oxidation of 2,3-dimethyl benzofurans. Chem. Ber..

[17] Zhang Q., Liu X.-T., Liang J.-Y., Min Z.-D. (2008). Chemical constituents from the stem of Caesalpinia decapetala. Zhongguo Tianran Yaowu.

[B18-molecules-17-01548] 18.See also the natural product discussed in references [19] and [20].

[19] Guo S., Tang Y.P., Duan J.A., Su S.L., Ding A.W. (2009). Two new terpenoids from fruits of Ziziphus jujuba. Chinese Chem. Lett..

[20] Chen H.-L., Wang L.-W., Su H.-J., Wie B.-L., Yang S.-Z., Lin C.-N. (2006). New terpenoids from amentotaxus formosana. Org. Lett..

[21] Tomioka H. (1997). Matrix isolation study of reactive *o*-quinoid compounds: Generation, detection and reactions. Pure Appl. Chem..

[22] Felix D., Winter C., Eschenmoser A. (1988). Fragmentation of α,β-epoxyketones to acetylenic aldehydes and ketones: Preparation of 2,3-epoxycyclohexanone and its fragmentation to 5-hexynal. Org. Synth. Coll. Vol..

[23] Chapman O.L., Chang C.C., Kolc J., Rosenquist N.R., Tomioko H. (1975). Photochemical method for the introduction of strained multiple bonds. Benzyne C ≡ C stretch. J. Am. Chem. Soc..

[24] Chiang Y., Gaplovsky M., Kresge A.J., Leung K.H., Ley C., Mac M., Persy G., Phillips D.L., Popik V.V., Roedig C., Wirz J., Zhu Y. (2003). Photoreactions of 3-diaz*o*-3*H*-benzofuran-2-one; dimerization and hydrolysis of its primary photoproduct, a quinonoid cumulenone: A study by time-resolved optical and infrared spectroscopy. J. Am. Chem. Soc..

[25] Voigt E., Meier H. (1977). About the photochemistry of heteroanalogue 3-diaz*o*-2-oxoindanes. Chem. Ber..

[26] Yoshioka E., Kohtani S., Miyabe H. (2011). A multicomponent coupling reaction induced by insertion of arynes into the C=O bond of formamide. Angew. Chem. Int. Ed..

[27] Yoshioko E., Kohtani S., Miyabe H. (2010). Sequential reaction of arynes via insertion into the π- bond of amides and trapping reaction with dialkylzincs. Org. Lett..

[28] Heaney H., McCarty C.T. (1970). Reactions of arynes with carbonyl compounds. J. Chem. Soc. D Chem. Commun..

[29] Heany H., Jablonski J.M., McCarty C.T. (1972). Aryne chemistry. XXXI. Reactions of arynes with α,β-unsaturated aldehydes. J. Chem. Soc. Perkin Trans. 1.

[30] Rayne S., Sasaki R., Wan P. (2005). Photochemical rearrangement of dibenzo [1,4] dioxins proceeds through reactive spirocyclohexadienone and biphenylquinone intermediates. Photochem. Photobiol. Sci..

[31] Müller E., Mayer R., Narr B., Rieker A., Scheffler K. (1961). Oxygen radicals. XVII. Dehydrogenation of bisphenols under formation of „inner“ spirocyclic quinol ethers. Liebigs Ann. Chem..

[32] Müller E., Kaufmann H., Rieker A. (1964). Synthesis of higher molecular weight compounds by phenol dehydrogenation. I. Oxidative trimerization of 4-methoxy-2,5-di-*tert*-butylphenol. Liebigs Ann. Chem..

[33] Hewgill F.R., Kennedy B.R. (1966). Oxidation of alkoxy phenols. VI. A hemiacetal from 4-methoxy-2-*tert*-butylphenol. J. Chem. Soc. C.

[34] Hewgill F.R., Hewitt D.G. (1967). Oxidation of alkoxyphenols. X. The reaction of 2,2'-dihydroxy- 5,5'-dimethoxy-3,3'-di-*tert*-butylbiphenyl with lead tetraacetate. J. Chem. Soc. C.

[35] Karpov V.V., Puchkov V.A., Khidekel M.L. (1968). Synthesis of spirocyclic trimers by the oxidative coupling of phenols in the presence of complex catalysts. Zhurnal Org. Khim..

[36] Karpov V.V., Khidekel M.L. (1968). Oxidative di- and trimerization during the oxidation of phenols by oxygen in the presence of copper-containing complexes. Zhurnal Org. Khim..

[37] Bowman D.F., Hewgill F.R. (1968). Radical coupling in the reaction of *o*-halophenols with base. Chem. Commun..

[38] Hewitt D.G. (1971). Phenol oxidation mechanism. Search for phenoxylium intermediates. J. Chem. Soc. C.

[39] Claus P., Schilling P., Gratzl J.S., Kratzl K. (1972). Preparation and oxidation of hydroxybenzyl alcohols. Monatsh. Chem..

[40] Becker H.D., Gustafsson K. (1976). Formation and photochemical isomerization of arylated 1,3- dihydr*o*-2*H*-azepin-2-ones. Tetrahedron Lett..

[41] Becker H.D., Gustafsson K. (1976). On the formation of spir*o*-substituted benzoxetes by phenol oxidation: preparation and valence isomerization of 3,3'-di-*tert*-butyl-5,5'-ditrityl-2,2'-dipheno quinone. Tetrahedron Lett..

[42] Becker H.D., Gustafsson K. (1977). Nucleophilic addition of amines to benz*o*-substituted oxetenes. Formation of 6-amin*o*-2,4-cyclohexadienones and their ring expansion. J. Org. Chem..

[43] Oleinik E.P., Mamysheva O.N., Gorbunova L.V. (1986). Characteristics of reactions of shielded germanium-containing phenols in the presence of oxygen under one-electron oxidation conditions. Doklady Akad. Nauk SSSR.

[44] Jamois D., Tessier M., Marechal E. (1993). Preparation of amphiphilic polyisobutylenes-β-polyethylen amines by Mannich reaction. II. Study of Mannich reaction on model systems. J. Polym. Sci. A.

[45] Müller E., Rieker A. unpublished work.

[46] Baltes J., Volbert F. (1955). Mechanism of antioxidant action. II. 3,3'-Di-*tert*-butyl-5,5'-dimethoxybi phenyl-2,2'-peroxide, a dehydrogenation product of 2-*tert*-butyl-4-methoxyphenol and its rearrangement to bis[4-methoxy-6-*tert*-butylbenzene-(2)]indigo. Seifen Anstrichmittel.

[47] Meier H., Schneider H.P., Rieker A., Hitchcock P.B. (1978). Sterically hindered “benzoxetes”-the first isolated oxepins. Angew. Chem. Int. Ed..

[48] Schneider H.P. (1981). Oxepines from o,o'-diphenoquinones, isomerization of α-ket*o*-*o*-quinone methides. Doctoral Dissertation.

[49] Schneider H.P., Winter W., Rieker A. (1978). Oxepines. Part V. The reaction of oxepino benzofurans with alcohols; the structure of Hewgill’s “trialkoxyspirobenzoxetes”. J. Chem. Res. Synopses.

[50] Hagen H., Becke F. (0120). BASF AG) Spiro[benzoxete-2,2'-imidazolidines] and corresponding hexhydropyrimidines. Ger. Offen. DE 2034758 A 1972.

[51] Aroyan A.A., Iradyan M.A. (1966). Synthesis of some tetrasubstituted ethylenediamines. Armyanskii Khimicheskii Zhurnal.

[52] Klosa J. (1966). Synthesis of spasmolytically active substances. XXI. Synthesis of α-alkoxybenzilic acid hydrazides. J. Prakt. Chem..

[53] Osman A.M., Bassiouni I. (1962). 2-Aryinaphthoxazoles and some other condensed oxazoles. J. Org. Chem..

[54] Mastagli P., Metayer M., de Bievre-Gallin G. (1948). Action of formamide on substituted benz aldehydes. Bull. Soc. Chim. France.

[55] Von Euler H., Adler E., Cedwall J.O. (1942). Reaction of phenols with C_2_H_2_. Arkiv. Kemi Mineral. Geol..

[56] Darapsky A., Berger H., Neuhaus A. (1936). Action of hydrazine hydrate on lactones. J. Prakt. Chem..

[57] Cox E.H. (1930). Mechanism and application of the Fries reaction. J. Am. Chem. Soc..

[58] Meier H., Issa A., Merkle U. (1979). 5a*H*-, 1Oa*H*-,15a*H*-Tribenzo[*b,f,j*,1,4,7]trioxa[9*b*]azaphenalenes- alledged benzoxetes. Z. Naturforsch..

[59] Voigt E., Meier H. (1976). Synthesis of the 2*H*-1-thiacyclobutabenzene system. Angew. Chem..

[60] Voigt E., Meier H. (1977). Photochemistry of heteroanalogue 3-diaz*o*-2-oxoindanes. Chem. Ber..

[61] Raasch M.S. (1980). Syntheses with halogen derivatives of thiophene and benzothiophene. J. Org. Chem..

[62] Schulz R., Schweig A. (1980). Theory and application of photoelectron spectroscopy. Part 87. Elucidation of thermal reaction by variable temperature photoelectron spectroscopy. A new synthesis of benzothiete and first direct evidence for transient benzothiete ketene. Tetrahedron Lett..

[63] Meier H., Mayer A. (1996). Synthetic equivalents for benz*o*- and naphthothietes. Synthesis.

[64] Kolmakov K.A., Kresge A.J. (2008). Synthesis of possible *o*-thioquinone methide precursors. Canad. J. Chem..

[65] van Tilborg W.J.M., Plomb R. (1977). Synthesis of benzothiete from benzo[*b*]thiophene 1,1-dioxide. J. Chem. Soc. Chem. Commun..

[66] van Tilborg W.J.M., Plomb R. (1977). Flash vapor-phase pyrolysis of thiophene 1,1-dioxides. Recl. Trav. Chim. Pays-Bas.

[67] Hortmann A.G., Aron A.J., Bhattacharya A.K. (1978). 3*H*-1,2-Benzodithiole oxides: Studies directed toward the generation of *o*-thiobenzoquinone methide and benzo[*b*]thiete. J. Org. Chem..

[B68-molecules-17-01548] 68.The pyrolysis zone can be filled with quartz particles for this purpose.

[69] Davis F.A., Awad S.B., Jenkins R.H., Billmers R.L., Jenkins L.A. (1983). Chemistry of sulfenic acids. 5. A novel rearrangement of 2,4,6-trineopentylbenzenesulfenic acid to 2-*tert*-butyl-4,6-dineopentylbenzo[*b*]thiete and 3,3-dimethyl-4,4-dihydr*o*-6,8-dineopentylbenzo[*b*]thiopyran. Synthesis of thiete sulfoxides. J. Org. Chem..

[70] Pohl E., Herbst-Irmer R., Sheldrick G.M. (1993). Structure of 4,6-bis(trifluoromethyl)-2,2-bis[2,4,6- tris(trifluoromethyl)phenylthio]-1-thiabenzocyclobutene. Acta Crystallogr. C.

[71] Hagen H., Amann A., Becke F. (4987). BASF AG) Spiro[benzothiete-2,2`-imidazolidines] and corresponding hexahydropyrimidines. Ger. Offen. DE 203.

[72] Hagen H., Amann A., Giertz H. (1978). (BASF AG) 2-(*o*-Alkylthiophenyl)-1,3-diazocycloalkene hydrohalides. U.S. Patent.

[73] Korobov M.S., Minkin V.I., Nivorozhkin L.E. (1975). Benzoid-quinoid tautomerism of azomethines and their structural analogs. XX. Imines of 5-nitrothiosalicylaldehyde. Zhur. Organ. Khimii.

[74] Goldfarb Y.L., Skorova A.E., Kirmalova M.L. (1966). Action of sodium in liquid ammonia on diethyl acetal of 6-methylthi*o*-3-methylbenzaldehyde. Izv. Akad. Nauk. SSSR Seriya Khim..

[75] Goldfarb Y.L., Skorova A.E., Kirmalova M.L. (1966). Action of sodium in liquid ammonia on 6- methylthi*o*-3-methylbenzaldehyde. Izv. Akad. Nauk SSSR Seriya. Khim..

[76] Nakayama J., Horikoshi R., Ishii A., Hoshino M., Kobayashi H. (1983). Reaction of benzyne with thiophosgene. Phosphorus Sulfur Silicon Relat. Elem..

[77] Okuma K., Shiki K., Sonoda S., Koga Y., Shioji K., Kitamura T., Fujiwara Y., Yokomori Y. (2000). Reaction of electronically stabilized thiones with benzyne. The isolation of thiobenzophenone- benzyne and thiopivalophenone-benzyne adducts. Bull. Chem. Soc. Jpn..

[78] Okuma K., Shiki K., Shioji K. (1998). Reaction of thiopivalophenones with benzyne. Formation of 2*H*-benzo[*b*]thietes. Chem. Lett..

[79] Meier H., Eckes H.-L., Niedermann H.-P., Kolshorn H. (1987). A nonspecific Diels-Alder reaction. Angew. Chem. Int. Ed..

[80] Meier H., Schmidt M., Eckes H.-L. (1989). Cycloaddition of benzothiete and 4-substituted styrenes. Chem. Ber..

[81] Meier H., Saul K., Jacob D. (1993). Reactions of benzothiete and imines. Liebigs Ann. Chem..

[82] Gröschl D., Mayer A., Schmidt M., Meier H. (1995). Synthesis of benzo[*b*]thioxanthenes. J. Prakt. Chem. Chem. Ztg..

[83] Meier H., Gröschl D. (1995). Reaction of 2*H*-1-benzothiete with diazo compounds in the presence of (II) rhodium acetate. Tetrahedron Lett..

[84] Gröschl D., Meier H. (1996). Cycloaddition reactions of 2*H*-benzo[*b*]thiete and conjugated cyclic dienes. J. Heterocycl. Chem..

[85] Gröschl D., Niedermann H.-P., Meier H. (1994). Cycloadditions of 2*H*-benzo[*b*]thietes and compounds with cumulated double bonds. Chem. Ber..

[86] Jacob D., Meier H. (1986). Cycloaddition reactions of benzothiete with azomethines. J. Heterocycl. Chem..

[87] Jacob D., Niedermann H.-P., Meier H. (1986). Cycloaddition reactions of benzothiete and hetero dienophiles for the synthesis of heterocyclic systems. Tetrahedron.

[88] Meier H., Saul K., Mengel R., Niedermann H.-P. (1991). Cycloaddition of benzothiete and oximes, oxime ethers and oxime esters. J. Heterocycl. Chem..

[89] Schmidt M., Meier H., Niedermann H.-P., Mengel R. (1990). 4*H*-3,1-Benzoxathiines from benzothiete and carbonyl compounds. Chem. Ber..

[90] Meier H., Gröschl D., Beckert R., Weiß D. (1997). Cycloaddition reaction of 2*H*-benzo[*b*]thiete and thiocarbonyl compounds. Liebigs Ann..

[91] Schmidt M., Meier H., Saleh S.-A. (1991). Cycloaddition of benzothiete and electron-deficient nitriles. J. Heterocycl. Chem..

[92] Saul K., Eckes H.-L., Jacob D., Meier H. (1993). Cycloadditions of benzothiete and aromatic nitroso compounds. Chem. Ber..

[93] Meier H., Schmidt M., Mayer A., Schollmeyer D., Beile B. (2012). Stereoselective synthesis of polycyclic thiopyrans. J. Heterocycl. Chem..

[94] Ohmo M., Kojima S., Shirakawa Y., Eguchi S. (1995). Heter*o*-Diels-Alder reaction of fullerene: Synthesis of thiochroman-fused C60 with *o*-thioquinone methide and oxidation to its S-oxides. Tetrahedron Lett..

[95] Kanakarajan K., Meier H. (1984). Ring enlargement of benzothiete to 2,3-dihydrobenz[d]isothiazoles. Angew. Chem. Int. Ed..

[96] Niedermann H.-P., Eckes H.-L., Meier H. (1989). Reactions of benzothiete with phosphorus- nucleophiles-A novel type of Arbuzov rearrangement. Tetrahedron.

[97] Eckes H.-L., Niedermann H.-P., Meier H. (1991). Addition of phosphorous nucleophiles to benzothiete. Chem. Ber..

[98] Meier H. (1996). Benzothiete, a versatile reagent in heterocyclic syntheses. J. Prakt. Chem. Chem. Ztg..

[99] Rokita S.E. (2009). Quinone Methides.

[100] Drohm C., Meyer H., Schweig A. (1995). Kinetics of a monomolecular photoreaction in a strongly light-absorbing solid solution exemplified by the forward reaction of the optically switchable *o*-thiobenzoquinone methide. Chem. Phys. Lett..

